# SVC: A Vision Transformer-based Spatially and Virtually Embedded Cell Model for Deciphering Subcellular Spatial Transcriptomic Heterogeneity

**DOI:** 10.64898/2026.10.07.757371

**Published:** 2026-10-14

**Authors:** Hui Wan, Penghui Yang, Siyu Hou, Jade Wang, Xiang Zhou

## Abstract

Subcellular transcript localization shapes gene regulation and cellular function, yet profiling it at scale remains difficult, limiting our ability to study subcellular heterogeneity across tissues and disease. Here, we present SVC, a Vision Transformer-based spatially and virtually embedded cell model that learns subcellular transcript organization within native tissue context directly from subcellular-resolution spatial transcriptomics. SVC represents each gene’s subcellular localization as an image patch and uses other genes within the same cell as context, enabling subcellular contextual learning of spatial dependencies that govern intracellular organization. By integrating gene-function priors, cell morphology, spatial context, and, when available, cell identity, SVC captures unified, spatially grounded representations across genes and cells that connect subcellular organization to cellular state and tissue microenvironment. Across diverse subcellular-resolution spatial transcriptomics datasets, SVC accurately predicts cell-specific subcellular transcript localization and co-organization, generalizes across tissues and technical platforms, and supports subcellular imputation of unmeasured genes and *in silico* perturbation. Leveraging these capabilities, SVC highlights disease-associated patterns of intracellular transcript reorganization and spatially heterogeneous responses to gene perturbations. Beyond prediction, SVC reveals systematic variation in subcellular transcript organization associated with gene function, cellular states, and tissue microenvironments, uncovering *de novo* spatial biology that is not accessible by cell-level analysis. SVC’s learned representations further transfer to cell- and tissue-level tasks, including cell-level expression imputation, cell clustering, and spatial domain detection. Together, SVC provides a general framework for modeling and interrogating subcellular transcript organization *in situ* , enabling scalable discovery of subcellular spatial biology.

